# Tumor suppressor role of miR-217 in human epithelial ovarian cancer by targeting IGF1R

**DOI:** 10.3892/or.2022.8415

**Published:** 2022-09-26

**Authors:** Jieyan Li, Dongmei Li, Weiyuan Zhang

Oncol Rep 35: 1671–1679, 2016; DOI: 10.3892/or.2015.4498

Following the publication of the above paper, a concerned reader drew to the Editors' attention that certain of the western blot data shown in Figs. 4B and 6D, and the scratch-wound assay data shown in Fig. 2D, were strikingly similar to data that had already appeared in previously published papers written by different authors at different institutes. Furthermore, there appeared to be anomalies associated with the cell invasion assay data portrayed in Fig. 2E. Having considered the issues identified, the Editor of *Oncology Reports* has decided that this article should be retracted from the Journal on account of the reappearance of previously published data and the uncertainties regarding the data in Fig. 2E. After consulting the authors, they agreed with the Editor's decision that this paper should be retracted. The Editor apologizes to the readership for any inconvenience caused.

